# Substantial changes in land and forest management led to critical transitions in peatland functioning over the last 700 years

**DOI:** 10.1038/s41598-025-02580-0

**Published:** 2025-05-25

**Authors:** Katarzyna Marcisz, Mariusz Bąk, Mariusz Lamentowicz, Piotr Kołaczek, Thomas Theurer, Paweł Matulewski, Dmitri Mauquoy

**Affiliations:** 1 Climate Change Ecology Research Unit, Adam Mickiewicz University, Poznan, Poland; 2 School of Geosciences, University of Aberdeen, Aberdeen, UK; 3 Anthropocene Research Unit, Adam Mickiewicz University, Poznan, Poland

**Keywords:** Palaeoecology, Palaeofire, Fire activity, Fire intensity, Monoculture, *Pinus sylvestris*, Fire ecology, Palaeoecology

## Abstract

Over the last 300 years, many European forests have been progressively modified toward monoculture ecosystems, with preference given to coniferous forests. These forests, often dominated by Scots pine (*Pinus sylvestris*), are currently impacted by various disturbance factors, e.g., more frequent windthrows, droughts, fires and insect infestations. Peatlands located in these monocultures are also significantly impacted, enhancing their vulnerability to drying and burning. Here, we investigate how the functioning of a *Sphagnum*-dominated peatland has changed during the last ca. 700 years along with the introduction of new forest management strategies–modification of a mixed-forest complex into a Scots pine monoculture. Multi-proxy, high-resolution palaeoecological analyses include AMS radiocarbon dating, pollen and spores, plant macrofossils, testate amoebae and historical data. Direct peatland fire disturbance was reconstructed using a wide range of charcoal analyses: charcoal counts and morphological types to reconstruct past fire activity, and Raman spectroscopy to reconstruct past fire intensity. The results obtained confirm that introduction of new management techniques impacted the functioning of the peatland, leading to critical transitions in vegetation composition and hydrology. Detailed analyses of a distinct charcoal layer present in the peat show that increased fire activity as recorded by charcoal accumulation does not necessarily equate to burning intensity. Therefore, we recommend the use of charcoal-derived wildfire intensity reconstructions in tandem with charcoal abundance studies.

## Introduction

The functioning of peatlands, once a common feature across the Northern Hemisphere, has been drastically impacted by human activity over decadal and centennial timescales^1,2^. As a result, many peatlands have been destroyed or severely disturbed, recording a gradual decrease in area across a number of countries^3^. For example, several studies from Europe assessing the number of wetlands and/or peatlands subject to significant disturbance or destruction have determined ca. 85%, 98%, and 94% of peatlands have been drained in Poland^4^, Germany, and the Netherlands^5^, respectively.

Most peatland disturbance originates with the ever-growing influence of human activity on the landscape. Such pressures may comprise direct or indirect actions, operating independently or in tandem, which in turn influence peatland ecosystem functioning. Direct anthropogenic pressures include peatland drainage^3,6,7^ or deliberate peat extraction (e.g., gardening, fuel, building materials^8–10^). Indirect impacts are often associated with the expansion of agriculture adjacent to the peatland^9,11^, or an implementation of changes in forest management techniques to the nearby forest complexes^12,13^. As a consequence, these disturbances most often perpetuate lowering of the water table, with a myriad of consequences for subsequent peatland functioning. By lowering the water table in a peatland ecosystem, the encroachment of surface vascular vegetation (e.g., dwarf shrubs, tree saplings) is often promoted, in turn, outcompeting mosses^14–16^. Pervasive hydrological disturbances lead to droughts and further increase the vulnerability of peatlands to fires and dramatic carbon losses^17–19^.

The impact of disturbances on peatlands and their subsequent regeneration have been studied thus far through observational and experimental studies^20–22^, and the use of palaeoecology^8,23–25^. Research to date has shown that regeneration efforts do not always lead to the reappearance of pre-disturbance vegetation^10^, and novel ecosystems may be created, possessing similar ecosystem functions but varying species compositions^26^. Hydrological conditions are crucial for peatland recovery^27,28^, including post-fire recovery^29,30^. Similarly, restoration is vital in protecting peatland carbon stocks and mitigating emissions^31,32^. There exists, however, a shortage of research regarding the impact of forest management on peatlands, specifically regarding complex interplays of multi-phase co-interactions of disturbance factors^33^. As most peatlands have been disturbed in the past by various factors, more attention needs to be paid to the understudied effects that local fire disturbance and vital large-scale forest structure modifications have had on *Sphagnum*-dominated peatland ecosystems.

**Fig. 1:**
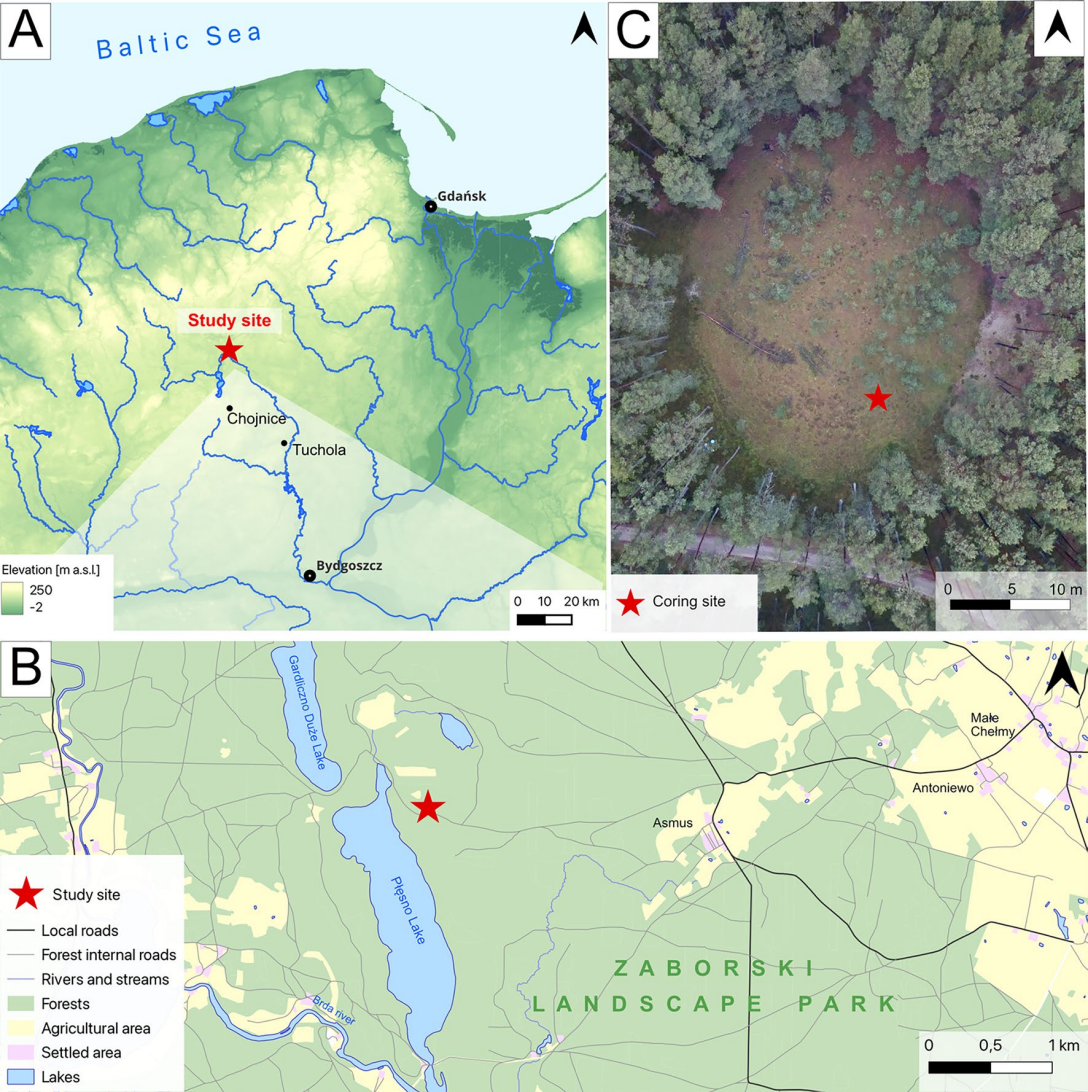
Study site location.

Our aim is to reconstruct how peatland ecosystem functioning has changed over the last ca. 700 years as a result of this unification in forest stands, in particular, the influence of forest management on peatland vegetation, hydrology, and fire activity. To achieve this, we have applied high-resolution contiguous sampling and multi-proxy reconstruction to an inter-forest peatland record, based on pollen and spores, plant macrofossils, testate amoebae, and a wide range of charcoal analyses (charcoal counts and morphological types, and Raman spectroscopy). We support our interpretations with historical and geopolitical data. We hypothesise that the establishment of monoculture forest has suppressed fire activity and stabilized hydrological conditions in the studied peatland (Fig. 1).

## Results and interpretation

### Chronology and peat and carbon accumulation rates

**Fig. 2:**
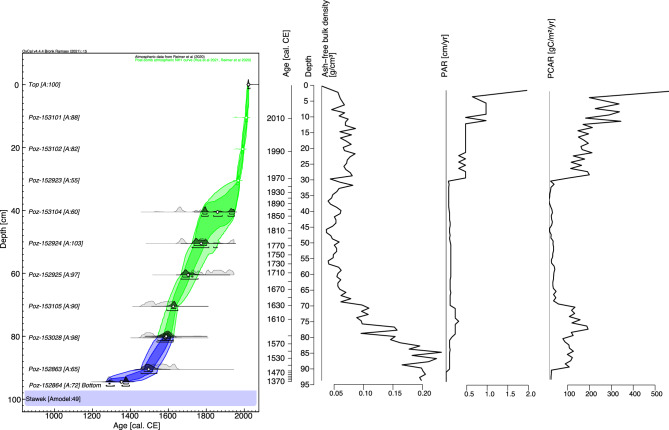
Age-depth model for the Stawek peat core and changes in bulk density and carbon accumulation rates.

The investigated sequence spans ca. 695 years between 1327 ± 60 cal. CE and 2022 cal. CE. The age-depth model revealed a model agreement index (A_model_) equal to 49% (Fig. 2) which is below the recommended minimum (60%; Bronk Ramsey^34^). However, we accepted this model as only one date (Poz-152923) revealed an individual date agreement to the model < 60%. All other dates have an individual agreement > 60%. The temporal resolution of samples was dependent upon their position in the peat profile. This ranged between 1–3 years/cm in the section between 0–30 cm (2022–1969 cal. CE) to 18–38 years/cm in the section between 91 and 96 cm (1478–1327 cal. CE). However, for the majority of the peat profile it did not exceed 11 years/cm. The 1 σ error of the modelled age ranged between ca. 0 and 60 years. The sediment accumulation rate (SAR) ranged between 0.03 and 0.06 (1478–1327 cal. CE) to 0.9 cm/year (2022–2011 cal. CE).

### Environmental changes in Stawek peatland

The Stawek record, as studied here, presents three distinct phases of peatland development across the study period, associated with fire activity (Phase 1), human occupation of the area (Phase 2), and land and forest management (Phase 3).

#### Phase 1 – Fire disturbance and post-fire regeneration (ca. 1400–1630 CE, 96–70 cm)

**Fig. 3:**
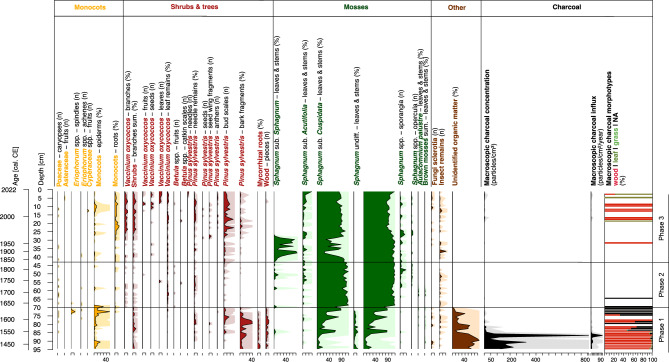
Plant macrofossil and macroscopic charcoal diagram (5 times exaggeration is marked).

Phase 1 in the development of Stawek peatland is associated with a prominent charcoal layer, visible in the core between ca. 1400–1580 CE (96–80 cm), characterised by a predominance of charcoal and *Substantia humosa* (i.e., highly decomposed organic material), exceeding 80% abundance in some layers (Fig. 3). Among the identifiable plant macrofossils, the peat was dominated by *Sphagnum* sub. *Cuspidata,* alongside other unidentifiable *Sphagnum* spp. Numerous monocot remains, fragments of *Pinus*, and wood were also present. Above the charcoal layer, between ca. 1600–1630 CE (80–70 cm), macrofossil evidence suggests a period of post-disturbance regeneration and a succeeding stabilisation of the peatland. This coincides with a fall in the proportion of *Substantia humosa*, a paucity of *Pinus* remains, and an increase in *Sphagnum* counts.

**Fig. 4:**
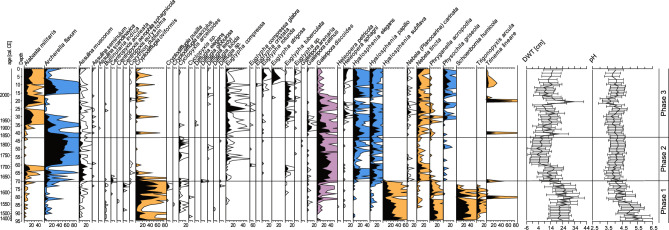
Testate amoeba diagram (5 times exaggeration is marked) and quantitative reconstructions of depth-to-water table (DWT) and pH. Dry indicator testate amoebae are marked in orange, wet indicators in blue, and disturbance indicator in purple.

Among testate amoebae present during this phase, the most common species include: *Cryptodifflugia oviformis*, *Hyalosphenia subflava*, *Phryganella acropodia*, *Schoenbornia humicola* and *Trigonopyxis arcula* (Fig. 4). The presence of *H. subflava* and *T. arcula* has previously been associated with fire/post-fire regeneration in moorland^35^, while *C. oviformis* and *S. humicola* are common in dry and disturbed habitats^36^, including within charcoal horizons and disturbed layers throughout other peat cores^37^. All aforementioned species incorporate mineral material from the environment to form their shells. A high abundance, as observed here, indicates increased mineral deposition into the peatland during this phase^23^. Notably, all of these species disappear from the testate amoeba communities at the end of phase 1, indicating a substantial environmental shift.

Quantitative reconstructions show that the mean depth-to-water table (DWT) in this phase equated to 23.2 cm (14.6–33.5 cm), with a sharp rise in water table depth from 22.2 cm to 14.6 cm in 1620 s CE. A mean pH value of 4.7 at this time, marked a fall from 5.6 early-phase, to 3.9 end-phase. (Fig. 4).

**Fig. 5:**
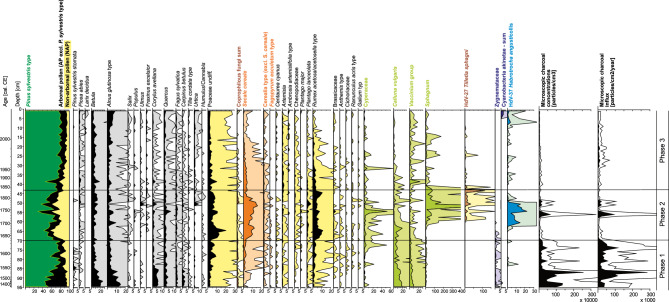
Palynological diagram including pollen, spores, selected non-pollen palynomorphs, and microscopic charcoal (5 times exaggeration is marked).

Pollen records suggest a closed canopy forest within the area at this time, dominated by *Pinus sylvestris* and an admixture of *Betula*, *Alnus glutinosa*, *Corylus avellana*, *Quercus*, *Fagus sylvatica* and *Carpinus betulus* (Fig. 5). At ca. 1535 CE (86.5 cm) a sharp drop in deciduous tree pollen – particularly *Betula*, *A. glutinosa*, *C. avellana* and *C. betulus* – accompanied a marked increase in *P. sylvestris* pollen frequency, exceeding 80% abundance toward the end of the phase. During this period, the loss of deciduous canopy supported an increase in cereal populations, primarily *Secale cereale*.

**Fig. 6:**
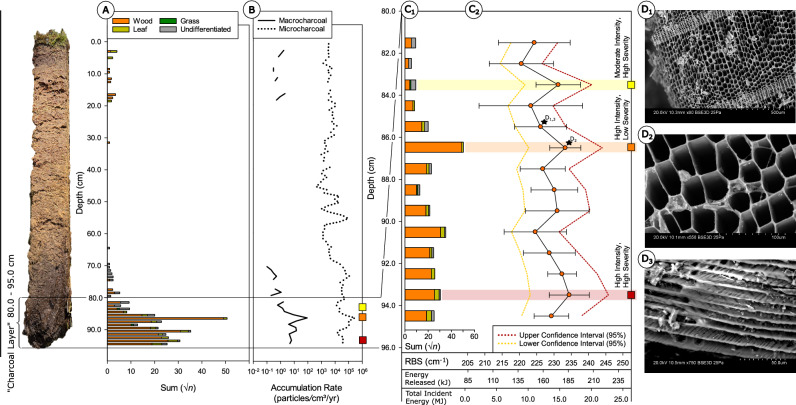
Detailed diagram presenting the Stawek charcoal layer: [A] macroscopic charcoal morphotype data (square-root transformed); [B] macro- and microscopic charcoal accumulation rates (logarithmic scale); [C1]—charcoal count and morphotype data with [C2] comparative results for the Raman spectroscopy of charcoal samples between core depths 80.0 – 96.0 cm. Values for median RBS are shown here alongside equivalent metrics for fire intensity reconstruction, including ‘energy released’ (kJ) and ‘total incident energy’ (MJ) after Eqs. (1) and (2), respectively^77^. Dotted lines indicate upper and lower confidence intervals (95%) for fire intensity reconstruction; and [D] SEM photographs of the largest found charred wood fragments of *Pinus sylvestris* presenting: [D1] wood cell structure, [D2] hyphae penetrating cell walls, and [D3] compression wood.

As mentioned previously, this phase in peat accumulation was dominated by charcoal, with overall abundance remaining very high up to ca. 1580 CE (81 cm). This is contrasted by a significant reduction in charcoal toward the end of this phase, between 1600–1630 CE (80–70 cm). Mean macroscopic charcoal influx (MAC) across this charcoal horizon totalled 11.3 particles/cm^2^/year, a considerable figure for peatlands (Figs. 3, 6), with peak MAC (89.9 particles/cm^2^/year) recorded at ca. 1530 CE (86.5 cm). With respect to charcoal morphology, most of the analysed macroscopic charcoal pieces were wood fragments, with a few leaves, and some indeterminable fragments (Fig. 6). Some of the charcoal pieces were large enough (> 1 cm) to permit species identification, all of which corresponded to burnt *Pinus sylvestris* (Fig. 6). Some charcoal pieces also displayed evidence of compression wood, commonplace in pines growing on peatlands^38^, as well as cell wall-penetrative fungal hyphae, suggesting one source of the charcoal included decomposing *Pinus* wood at the peatland surface^39^. This is further evidence for localised *Pinus* encroachment and peatland drying during this phase.

In contrast, mean microscopic charcoal influx (MIC) across this layer totalled 59,107 particles/cm^2^/year, demonstrating substantial regional fire activity (Figs. 5, 6). Coinciding peaks in MAC and MIC (285,309 particles/cm^2^/year) at ca. 1535 CE (86.5 cm) suggest the representation here of a single or several fire events, or that fire activity was commonplace throughout the entire region at this time. Regional fires represented by MIC were still active during the regeneration phase of Stawek peatland (ca. 1600–1630 CE, 80–70 cm depth), with a mean influx of 44,005 particles/cm^2^/year.

Randomised macrocharcoal pieces, isolated between 81.0 and 95.0 cm (ca. 1580–1370 CE), offer median Raman band separation (RBS) values and equivalent wildfire intensity metrics (i.e., energy released and total incident energy) indicative of a general trend in fire intensification with depth (Fig. 6). This intensification appears to occur with an approximate cyclicity, as peak intensification occurs every ~ 3–4 samples (1.5–2 units). Troughs in intensification immediately follow periods of cyclical peak intensity, suggesting a ‘rapid’ reversal of fire-promotive conditions. Broadly, wildfire intensity as reconstructed here shows an inverse relationship with standard deviation at each interval, suggesting an increase in sample variability (i.e., fire system variability) during periods of low intensity. This may reflect a variability in the degree to which material is charred, due to increased material preservation under lower fire intensity conditions (i.e., less material is combusted away). This coincides with an observation of brown colouration, indicative of partial charring in samples of lower median RBS (e.g., STA_81.5). Partial charring is also evident in poorly resolved D- and G-bands, with spectra that exhibit an increased distance between the horizontal axis and the righthand tip of the spectrum. This feature is representative of ‘fluorescence angle’ and typically denotes heterogeneous physiochemistry (e.g., lignocellulosic^40^) that has not been removed fully under progressive charcoalification^41^. This variability may also be related to the dominant or constituent mix of fuels charred, and their inherent physiochemical differences, in turn responding differently to wildfire conditions.

#### Phase 2 – Thriving agricultural activities (ca. 1630–1840 CE, 70–43 cm)

*Sphagnum* abundance increases (Fig. 3), peaking at almost 100% between ca. 1645–1685 CE (67.5–63.5 cm depth). In this instance, the dominant *Sphagnum* species were those from sub. *Cuspidata*, however, some leaves and stems of *Sphagnum* sub. *Acutifolia* and sub. *Sphagnum* were also present, alongside remains of vascular plants, mainly those of *Vaccinium oxycoccos* and *Pinus sylvestris*. The domination of *S*. sub. *Cuspidata* points to acidification and formation of lawn microforms typical for poor fens.

The testate amoeba community composition also changed in this phase (Fig. 4), with the replacement of fire-related taxa by common dry indicator species, for example *Alabasta militaris* and *Nebela tincta*. At the same time, *Galeripora discoides* appeared in greater numbers–a species that is common in hydrologically unstable habitats^36,42^. It was soon followed by an encroachment of wet indicator mixotrophic taxa, namely, *Hyalosphenia elegans* and *Hyalosphenia papilio*.

At the beginning of the eighteenth century CE, wet indicator species *Archerella flavum* dominated the recorded testate amoeba communities, coinciding with quantitative DWT reconstructions and an indication of a substantial rise in water tables. A mean DWT value of 8.5 cm (16.9 cm to 2.2 cm) was accompanied by a sharp increase in depth-to-water table from 11.9 cm to 6.2 cm at the beginning of eighteenth century CE (ca. 1700–1715 CE, at 60.5–59.5 cm depth). Additionally, pH values ranged between 4.2 and 3.8 (mean pH: 4.0) which indicates high acidity in the peatland at this time.

Pollen analysis indicates a change in local vegetation composition and an opening of the landscape (Fig. 5), represented by a substantial drop in arboreal pollen (‘AP’ *herein*) – falling to 55% at ca. 1670 CE (64.5 cm; Fig. 5). Increases in Poaceae (≤ 20%), *Rumex acetosa/acetosella* type (≤ 11%) and crops, principally *S. cereale* (> 16%), indicate deforestation and a rapid increase in agricultural activity within the study area at this time^43^. Relative abundance of *S. cereale* remained elevated for much of phase 2, up to ca. 1800 CE (47.5 cm), later falling below 4.5%.

A particularly low abundance of macroscopic charcoal preserved during this phase suggests little to no local fire activity (Fig. 3), or an intrinsic constraint on charcoal preservation. The absence of evidence for fire activity suggests successful suppression efforts by local populations, during active management of the land in the region. With respect to regional fire activity, two peaks in MIC – 94,139 (55.5 cm) and 20,500 (48.5–50.5 cm) particles/cm^2^/year, at ca. 1740 CE and 1770–1790 CE, respectively – coincide with short-term drops in AP indicative of deforestation.

#### Phase 3 – The establishment and impact of Pinus monoculture (ca. 1840–2022 CE, 43–0 cm)

The top section of the Stawek core presents as *Sphagnum*-dominated peatland, comprised primarily of *S.* sub. *Cuspidata*, with a short-term dominance of *S.* sub. *Sphagnum* between ca. 1885–1970 CE (37.5 and 29.5 cm), and the presence of *S.* sub. *Acutifolia* (Fig. 3). A large number of vascular plant remains were also present, mainly those of *V. oxycoccos* and various fragments of *P. sylvestris*. The presence of *S*. sub. *Sphagnum* may indicate a change in the microtopography of the peatland surface, specifically to the formation of hummocks with dwarf shrubs common for ombrotrophic peatlands.

Testate amoeba compositions during this phase were characterised by a fall in the abundance of *A. flavum*, while *A. militaris* and *N. tincta* became more abundant (Fig. 4). Within the final 20 years of accumulation, the abundance of *G. discoides* fell, alongside a re-appearance of *A. flavum* in greater numbers, together with the mixotrophs *H. elegans*, *H. papilio* and *Physochila griseola*. Quantitative reconstructions indicate a substantial rise in water tables, including a mean DWT value of 12.8 cm (21.3–4.9 cm). Two transient periods of falling water tables at ca. 1860 CE and 1990 CE (40.5 and 20.5 cm, respectively) correspond to the appearance of *Trinema lineare* and the re-appearance of *C. oviformis* as small, dry habitat indicators^44^. The increase in DWT at ca. 1860 CE is similarly related to a change in dominant *Sphagnum* species under the encroachment of *S.* sub. *Sphagnum*. Moderate drying can be observed in the uppermost peat layers, and pH values range between 3.5 and 4.3 (mean pH: 3.9), indicating continued high acidity.

In this phase, pollen compositions suggest an ecosystem dominated by trees, supported by a peak in AP abundance of 95% in the 2010s, and a mean AP value for this phase of 90.5%. Forest composition was, at this time, comprised primarily of *Pinus* and an admixture of *Betula* and *A. glutinosa* (Fig. 5). A reduction in the abundance of Poaceae, cereals and ruderal taxa such as *Rumex acetosa/acetosella* type indicate reforestation of the area, with a substantial decrease in localised agricultural activities.

Fire activity was very low in this phase (Figs. 3, 5), with only a few small, charred wood fragments and leaves present in this phase of peat accumulation. Charcoal counts remained low (mean MAC: 0.2 particles/cm^2^/year), indicative of limited (if any) localised burning, with some evidence of regional burning (mean MIC: 4269 particles/cm^2^/year). Microcharcoal accumulation rates at this stage are, however, lower than any other period through the Stawek profile, raising questions as to the true extent of regional fire activity.

## Discussion

### Management practices as a proponent of critical transitioning in peatlands

Numerous natural and anthropogenic factors substantially impact peatland functioning, leading to modifications in various peatland ecosystem components, including vegetation, microbial food webs, hydrology, peat/carbon accumulation rates, and others^1,27^. The stronger and more pervasive the disturbance, the greater its impact on peatland functioning^19^. Many examples show critical transitions in wetlands as a source of transformation in certain ecosystems–modifying their functioning due to the crossing of tipping points^27,45^. In the Stawek record, we have distinguished two critical transitions in ecosystem functioning, visible in plant macrofossil, testate amoeba, and charcoal data. By correlating high-resolution pollen and historical data, we have further identified the causes and effects of these substantial changes in peatland functioning.

At the onset of the seventeenth century CE, the first critical transition in Stawek is marked by a sharp change in the sediment type–from highly decomposed peat, rich in charcoal, to the appearance of *Sphagnum* and heightened peat accumulation rates. Between the fourteenth and seventeenth centuries CE this peatland experienced a subsequent drying with surface fire activity, as evidenced by the analysis of charcoal particles and the presence of hyphae in the wood cells (Fig. 6). Though substantial at that time, the frequency of fire activity during this period is indistinguishable with contemporary methods in charcoal analysis. An abrupt reduction in fire activity, as observed, is possibly attributable to the activity of local communities present in the area at the time, utilising this region for agricultural purposes.

An increase and decrease in cereal and arboreal pollen, respectively, implies the opening of forest canopies with moderate rapidity, expansion of farming, and suppression of natural fire activity. It is likely that a number of the broadleaf species (e.g., oak, hornbeam) were used by local communities to establish villages and households–well documented in Polish archaeological and palaeoecological studies^46,47^. The opening of the forest and the establishment of agricultural land at this time is further observed in the sharp increase in cereal pollen types typically grown in this region (e.g., *Secale cereale, Fagopyrum*), often coupled with the drainage of wetlands^33^. The expansion of farming and intensification of land use, as observed here, represent ‘tipping points’ in peatland development, recorded previously, e.g., in Pawski Ług peatland, where in the mid-fourteenth century a rapid transition from a lake to a *Sphagnum*-dominated peatland took place^12^. In the case of Pawski Ług, significant alterations to land management were brought about by the Knights of the Order of St. John (Joannites), who controlled estates in Brandenburg and Pomerania. Consequently, German settlers were introduced to the former Slavic territories in 1350^12^. The introduction of a feudal economy on less-developed lands centred around landscape opening for the establishment of agriculture and was a common practice in the Middle Ages across Europe^47–50^.

The landscape opening around Stawek peatland was later facilitated by more invasive activities related to the growing economy. In the seventeenth and eighteenth centuries CE, tar and glass factories were principal industry sectors in the region, from which goods were exported to various European locations until the 1^st^ World War^51^. Charcoal, produced in numerous hearths across the Tuchola Forest, represented another significant export commodity^51^. Two temporary reductions in cereal pollen abundance in the eighteenth century CE may correspond to periods of civil and political unrest, e.g., the ‘Third Northern War’ (1700–1725) or the ‘Seven Years War’ (1756–1763), the effects of which have been noted in the pollen record of Lake Czechowskie (Northern Tuchola Forest)^52^. This perturbation in cereal pollen is similarly noted with the first partition of Poland in 1772, and the annexation of Pomerelia (incl. the Tuchola Forest) to Prussia^33,53,54^.

A contrasting process marks the second critical transition, namely, the closing of the forest and plantation of pine within the Stawek study area. This abrupt change in forest management strategy was the result of Prussian administrative decisions, as defenders of land inclusive of the Tuchola Forest, subsequent to the partition of Poland in 1772. In the first decades of the Prussian administration, private lands were still used by peasants for agriculture^55^. However, the liquidation of state forests had declined in the 1830s, and ceased in 1860^56^. The introduction of new management strategies focussed on timber production, resulting in a substantial modification of forest composition. This was characterised by a shift from a 40% broadleaf to 60% Scots pine mix to almost 100% *Pinus sylvestris* forests^24,33^.

According to historical sources, by as early as 1893, *Pinus* had been planted in up to 99% of forests in the Tuchola Forest District^33,54^. Moreover, the *Pinus sylvestris* saplings planted during this period were non-native, transported from Germany (primarily Schwarzwald) and thus ill-adapted to the local climatic and edaphic conditions. Subsequently, German *Pinus sylvestris* have been gradually replaced with native Polish *Pinus sylvestris* saplings, to strengthen the forest stands (Local Forester, *personal communication*).

This change in forest composition was also associated with a division of the forest into clear-cut, squared/rectangular districts. Domination of coniferous species increased soil acidity, creating a so-called “sea-salt effect”^54,57,58^. Management by Prussians involved not only a substantial change in forest stands, but also water management, characterised by the introduction of melioration and drainage and, in consequence, the drying of various types of wetlands^51^ (see Bąk, et al.^54^ for a detailed historical background). Deliberate lake and pond desiccation, and progressive soil acidification, created a suitable acid habitat for the encroachment of *Sphagnum* and finally the terrestralisation of water bodies, still observed today^59–62^. Bagno Stawek Nature Reserve offers another appropriate analogue, located ca. 500 m from our study site. This alkaline fen, possessing endangered flora such as *Saxifraga hirculus*, *Cinclidium stygium* and *Paludella squarrosa,* is now suffering from *Sphagnum* encroachment (personal observation), leading to its gradual degradation.

The closure of forest stands is visible in the Stawek palynological record, and a highly acidic environment is evident in both plant macrofossil and testate amoeba records (Figs. 3, 4, 5). The presence of diverse *Vaccinium oxycoccos* macroremains and an increase in abundance of *S.* sub. *Sphagnum* points to ombrotrophication and formation of hummock-hollow microtopography on the surface of the peatland. It is evident that the introduction of monoculture led to minor hydrological disturbances in the peatland but did not significantly impact its acidity–most likely due to the influence of *Pinus sylvestris*. A similar process was observed in the Noteć Forest in north-western Poland, which has a similar history to the Tuchola Forest – it was annexed into Prussia (1772) and modified to a *Pinus sylvestris* monoculture^63^. These actions resulted in an acidification of the area, lowering of the water table, and the encroachment of *Sphagnum* into existing water bodies, thus creating many new peatlands. This transformation is best represented by the *Sphagnum* encroachment and colonisation of Lake Rzecin, currently forming the largest peatland in the Noteć Forest^63,64^.

Monoculture forests, as unified forest stands dominated by one tree species, are notably susceptible to various disturbances, such as droughts, forest fires or insect infestations^33,65–67^. Surprisingly, however, there has been no observable increase in fire activity (*as per* the charcoal record) across the Tuchola Forest within the last 200 years. Though few forest fires have been reported within historical sources, these were located elsewhere in the forest complex, and seemingly had no influence over the studied peatland area.

The Tuchola Forest also experienced several insect outbreaks (mainly caused by *Panolis flammea* and *Lymantria monacha* that feed on *P. sylvestris*) in the twentieth century, particularly in the years 1922–1924, 1962–1963 and 1978–1982^24,33,53^. Again, there was no discernible record of this disturbance, typically characterised by insect remains in the macrofossil record. This may be countered, only, by a minor decrease in *P. sylvestris* abundance, visible in the pollen record during the 1980s (Fig. 5). A similar palynological signal was recorded in the Martwe peatland, ca. 50 km from Stawek^24^.

### Evaluating multi-proxy trends in fire activity and intensity

Among high-resolution peatland charcoal records, studied in northern Poland over the last decade (e.g^6,12,13,24,68–72^.,, only Stawek peatland, as investigated here, and Głęboczek (also located in the Tuchola Forest) possessed distinct and substantial charcoal layers (^37,73^; Fig. 6).

Relative to Stawek, the Głęboczek charcoal layer was substantially thinner and its presence was associated with a ca. 500-year hiatus^73^. Whilst radiocarbon dating and age-depth modelling offer no evidence of significant inversion in the Stawek peat record (Fig. 2; Marcisz, et al.^37^), it may be fair to assume that a number of short-term hiatuses, undetectable by sediment dating, are present. This offers a considerable opportunity to investigate the Stawek charcoal layer at high resolution, and determine the correlation, if any, between several complementary fire proxies, including Raman spectroscopy as a novel method in fire intensity reconstruction^74–77^.

It is apparent under preliminary study that, as a result of heightened charcoal volume and proportions of highly decomposed peat (determined as *Substantia humosa*, Fig. 3), it is not possible to identify plant macrofossils in the bottom charcoal layer, excluding a few *Sphagnum* fragments. Reconstructed water tables were low (< 20 cm), and almost no mixotrophic testate amoeba species were present, suggesting that the site was not highly acidic. An increase in charcoal quantity from the base of the peat profile to 86 cm (ca. 1535 CE) further coincides with an increase in NAPs. Furthermore, immediately following the layer in which the highest charcoal sum was recorded (86.5 cm) we observe a substantial decrease in AP and deciduous pollen taxa: *Alnus, Corylus, Quercus* and *Carpinus*. It may be assumed, therefore, that these were selectively harvested by local populations to obtain timber for housing and/or everyday use^46^.

Larger charcoal pieces (> 1 cm) found within this layer were all identified as *Pinus* fragments, burned when the trees were dead and decomposing, as indicated by the presence of hyphae in the charcoalified wood cells^39^ (Fig. 6). This suggests that pine trees burnt in situ and confirms that the peatland was dry in this fire period, facilitating the burning of surface biomass that may have otherwise been waterlogged under wet peatland conditions. Dead *Pinus* wood situated on the surface of the peatland likely produced a considerable amount of charcoal and formed this layer.

Above the layer with the highest charcoal sums, the record indicates a simultaneous decrease in charcoal and an increase in cereal pollen, indicative of a reduction in fire activity and intensification of agricultural activities in the region. At the same time, *Pinus* pollen sums increase, in this instance, a likely artefact of high pollen production by *Pinus* rather than an expansion in *Pinus* extent. Therefore, it may be assumed that deciduous trees were selectively felled to obtain wood, the forest was opened to establish agricultural space, and local populations suppressed natural fires.

Several methods have been applied across the field of palaeoecology to reconstruct past fire intensity. For example, the trait approach has been used to group tree species into various fire trait categories (e.g., resister, avoider), with an assumption of past fire intensity predicated upon pollen sums and traits associated with certain species^78^. However, this method is limited by the potential for a species to burn in low and high-intensity fires, irrespective of fire resistance, assuming fire is present in the natural environment. Therefore, species composition alone cannot unequivocally inform about the level of fire intensity. Similar caution is necessary for interpretations based on the morphology of fossil charcoal fragments (e.g., length-to-width ratio), as applied to fire intensity reconstructions from ocean and lake sediments (e.g^79,80^.,,). The morphological and morphometrical features of individual charcoal fragments can be strongly altered by taphonomic processes during deposition as well as chemical processing of the sediment and sieving during sample preparation. The assumptions as to what morphotype of charcoal is produced in certain environments may, therefore, be inaccurate and a simplification of otherwise complex fire behaviour in various ecological systems.

A range of factors influence the amount of charcoal produced during wildfires. Fuel types are important, as charcoal production varies between^81^ and within a range of plant species^82^. In woodlands, if abundant understorey tree saplings are present a wildfire will produce more charcoal compared to a stand with fewer saplings. Saplings are highly susceptible to combustion due to their small diameter stems and thin bark (bark thickness is an important trait controlling the potential for charcoal production). Fine fuels (graminoids, leaves, needles, ferns, mosses and small twigs) with low fuel moisture are likely to record the opposite response, as charcoal is more likely to be consumed into ashes and gases during combustion. Given this, it is simplistic to assume a direct correlation between the number, volume and/or concentration of charcoal fragments, and fire intensity (e.g^83,84^.,,). Whilst the hypothesis ‘more equals more’ may seem intuitive, contemporary observations in the experimental replication of wildfire suggest higher intensity fires consume (i.e., combust) a greater proportion of fuel mass at a higher rate, though this remains dependent upon the characteristics of the fuel (e.g^85,86^.,,). As a result, the available fuel for preservation as charcoal is limited. Hence, less charcoal should be preserved in the sediment/peat resulting from combustion under high intensity fires, and vice versa. Similar assumptions are made under calculations of ‘Fire Radiative Power’ (FRP), as determined by a transfer function model^87^ whereby FRP is calculated from charcoal sums. This suggests a close association between charcoal sums and FRP^69,88^that may not, in fact, reflect true fire intensity as reconstructed here by application of Raman spectroscopy. This has been emphasised in further studies, including the application of a variety of methods to reconstruct fire intensity in sediment records (e.g^89^.,) and via Fourier Transform Infrared spectroscopy (FT-IR)^90–94^.

Raman spectroscopy, applied to the physiochemical analysis of wildfire charcoals, represents a rapid, versatile, and non-destructive method of palaeofire intensity reconstruction^74–77^. In studying changes to nanoscopic crystalline structures within experimental charcoals, generated through laboratory pyrolysis at increasing temperatures, trends in spectral band-derived parameters have formed a robust basis for thermometry. The application of experimental pyrolysis in this instance yields ‘pyrolysis intensities’ as an approximate measure of wildfire intensity^95^. However, the temperature of pyrolysis has limited applicability in understanding the complex energy fluxes that occur during flaming combustion as experienced in a natural wildfire^96,97^. Recent efforts have, instead, developed measures of true wildfire intensity from calorimetric experimentation and Raman spectroscopy in tandem, considering the combined role of pyrolysis, flaming combustion, residual heating, and char oxidation in modifying the physiochemistry of charcoal^77^. From this, accurate reconstructions of fire intensity, as a function of energy release, are possible over different timescales, consistent with modern methods in wildfire characterisation^98^.

The application here of Raman spectroscopy to Stawek charcoals suggests no definitive correlation between Raman-derived fire intensity and charcoal sums. Samples from which the most intense fires have been reconstructed were seldom associated with the greatest sum of charcoal (Fig. 6), countered only by measurements at 86.5 cm (ca. 1535 CE). Instead, samples at depths 83.5, 93.5, and 92.5 cm (ca. 1560, 1405, and 1440 CE, respectively) presented reduced charcoal sums alongside high intensity fires. This suggests instances of an inverse relationship between charcoal sum and Raman-derived intensity, as previously hypothesised. This is supported by samples at 90.5 cm depth (ca. 1500 CE), recording the second-highest charcoal abundance and low fire intensity. It is apparent, however, that this association is inconsistent. As a result, the comparison of charcoal abundance and reconstructed intensity may yield further insight into the nature and behaviour of past fire activity, specifically as a measure of fuel consumption, ecosystem disturbance, and fire severity^97^. For instance, reduced charcoal abundance coinciding with high-intensity fire reconstructions may represent pervasive, high-intensity localised burning. This would likely result in the consumption of greater masses of available fuel, and a reduction in charcoal production. In contrast, high-intensity fires recorded alongside high charcoal sums may indicate ephemeral fire events. Dependent upon the intended purpose and outcomes of Raman-derived palaeofire reconstructions, intensity reconstructions are therefore recommended in tandem with charcoal abundance studies. This research, however, reiterates the inapplicability of charcoal abundance studies as a sole measure of fire behaviour.

## Conclusions

This study focused on a high-resolution, multi-proxy reconstruction of environmental change within a *Sphagnum*-dominated peatland, affected by seven centuries of substantial change in land and forest management strategies. Two critical transitions, recorded in the peat profile, were related to an introduction of agriculture and modification of forest stands from mixed-forest to coniferous monoculture. A rapid development of agricultural activities–as confirmed by palynological, plant macrofossil and testate amoeba data–resulted in a reduction in fire activity, perpetuating the dominance of *Sphagnum* and an increase in peat accumulation rates. Further afforestation and the establishment of *Pinus* monoculture impacted the peatland, stabilizing *Sphagnum* growth and acidity levels.

Peatland fire disturbance, reconstructed using a wide range of charcoal analyses, has given us an insight into a number of key fire characteristics. We show that increased fire activity, as recorded by charcoal morphotypes and accumulation rates, does not necessarily equate to Raman-derived burning intensity. Indeed, the relationship between charcoal counts and thermometric intensity reconstructions can instead provide essential insight into fire severity, for which no direct proxy exists to date. We therefore recommend the use of charcoal-derived intensity reconstructions in tandem with charcoal abundance studies for greater accuracy and insight when reconstructing and interpreting peatland fire records. These results can be highly useful for the development of accurate future multi-proxy reconstructions of fire activity and the interpretation of past fire regime changes, especially debunking simplified interpretations of charcoal sums as direct evidence for high-intensity fires. Moreover, as ongoing efforts in peatland conservation and restoration under anthropogenic climate change are becoming more challenging for foresters and forest managers, we are convinced that the results of this study will underline the necessity of wetland protection. Priority should be given to protecting peatland hydrology because high water tables provide water retention and fire protection for not only peatlands, but also the neighbouring forest, at the same time protecting carbon stored in peat for millennia.

## Methodology

### Study site

The studied site–Stawek peatland–is located in the Tuchola Forest, a *Pinus sylvestris* monoculture forest located in Northern Poland (53°53′22′′N, 17°33′06′′E, 138 m a.s.l.; Fig. 1). Stawek is a small (< 1 ha) *Sphagnum*-dominated kettle-hole peatland, encompassed by birch and pine to the north, with numerous collapsed dead trees on the surface^37^. The study area is located close to the Pomeranian ice margin of the Vistulian Glaciation, dated to ca. 17,000–16,000 cal. BP^99^. The Tuchola Forest thereby represents a young glacial landscape covered by till and sandur, rich in glacial landforms originating from the melting of dead ice^100^, including depressions, within one of which Stawek peatland formed.

The Tuchola Forest represents one of the largest forest complexes in Poland, covering an area of ca. 300,000 ha^101^. Historically, this region was populated by Scots pine-dominated (*Pinus sylvestris*) mixed forest, as an admixture with many deciduous taxa such as birch (*Betula pendula*, *B. pubescens*), alder (*Alnus glutinosa*), hornbeam (*Carpinus betulus*), beech (*Fagus sylvatica*), oak (*Quercus robur*) and hazel (*Corylus avellana*)^24,102,103^. Within the last six centuries, the region encompassing the Tuchola Forest has experienced complex political change, including administrative affiliations^33,104^. Following the accession of this region to Prussia at the end of the eighteenth century, administrative decisions made by successive governments included the modification and repurposing of these forests into a Scots pine monoculture^33,53,104^. A detailed description of these forest modifications due to administrative decisions can be found in Bąk, et al.^54^.

The study area is characterized by a transitional climate, the coldest and warmest months comprising January (− 1.7 °C) and July (18.2 °C), respectively, while annual precipitation averages 699 mm (mean values for the 1991–2021 period recorded at Swornegacie village located ca. 5 km from the site^105^).

### Fieldwork

The choice of the site was determined by a previous study performed from the Stawek peatland where we reconstructed environmental disturbances in the deeper peat layers (90–110 cm) using the multi-proxy approach, exploring the potential of neodymium isotopes in peat as a past local disturbance proxy^37^. The coring campaign in 2020 (survey included several coring locations across the site using a gouge auger and sampling of a peat core using a small Instorf corer – 50-cm long with 5 cm diameter chamber) revealed distinct charcoal layers present in Stawek peat which we decided to explore in detail. A single one meter-long undisturbed peat monolith utilised in this study was extracted in April 2022 with a Wardenaar sampler (100 × 10 × 10 cm^106^) from the centre of Stawek peatland. This time we sampled with a larger corer to obtain more material for multi-proxy analyses. Even though investigations based on a single core are standard practice in palaeoecological research^107^, we are aware of their limitations, specifically when it comes to drawing broad conclusions related to the spatial variability of peat layers. The obtained peat monolith was packed into a plastic tube and wrapped in plastic to ensure minimal disturbance and contamination during transport.

### Laboratory work

In total, 91 contiguous peat sub-samples were obtained at 1 cm resolution (2 cm resolution was applied to the uppermost 8 cm, an undecomposed *Sphagnum* layer) across a 95 cm undisturbed peat monolith. Multi-proxy analyses applied to all of the 91 sub-samples included: bulk density, peat and carbon accumulation rates, pollen and spores, plant macrofossils, testate amoebae, and charcoal analyses.

### Radiocarbon dating and age-depth modelling

A Bayesian age-depth model was constructed to determine the absolute chronology for the profile based upon 10 ^14^C AMS dates (Table 1). The age-depth model was constructed using the *P*_*Sequence* function in the OxCal 4.3 software with parameters: *k*_*0*_ = 0.5, *log*_*10*_*(k/k*_*0*_*)* = 1 and *interpolation* = 0.5 cm^34,108,109^. The IntCal20^110^ and Bomb21NH1^111^ ^14^C atmospheric curves were used as the calibration sets. All dates were included in the model. The sections of the profile with potential changes in the accumulation rate of deposits (AR_deposits_) were introduced to the model as boundaries (*Boundary* command). Based on the stratigraphy, we placed the boundaries at depths of: (i) 96 cm—the base of the model, (ii) 80 cm—the boundary between a decomposed layer rich in charred fragments of plants (below) and undecomposed peat, and (iii) 0 cm—the top of the profile. In the following text mean (*μ*) values of modelled dates were rounded to the nearest decade. The ages are expressed as ‘CE’ (Common Era). The AR_deposits_ was calculated using OxCal 4.3 software and presented as cm yr^−1^.

**Table 1:** ^14^C Radiocarbon dates for the Stawek profile.

No	Laboratory code –sample number	Depth (cm)	^14^C date (^14^C BP)	Calibrated dates [cal. CE (2σ–95.4%)	Dated material
1	Poz-153101	10.5	104.25 ± 0.32 pMC	1952–1958 (12.9%) 2006–2014 (82.5%)	*Sphagnum* stems
2	Poz-153102	20.5	114.32 ± 0.38 pMC	1957–1962 (10.0%) 1986–1996 (85.5%)	*Sphagnum* stems, charcoal
3	Poz-152923	30.5	154.53 ± 0.4 pMC	1964–1974 (95.4%)	Vascular plant remains
4	Poz-153104	40.5	225 ± 30	1636–1688 (41.2%) 1730–1806 (47.6%) 1925–… (6.6%)	Vascular plant remains
5	Poz-152924	50.5	195 ± 30	1646–1695 (24.1%) 1724–1812 (54.0%) 1838–1844 (0.5%) 1853–1877 (1.3%) 1916–… (15.5%)	Vascular plant remains
6	Poz-152925	60.5	140 ± 30	1672–1778 (37.2%) 1798–1944 (58.3%)	Vascular plant remains
7	Poz-153105	70.5	345 ± 30	1470–1637 (95.4%)	Charcoal
8	Poz-153028	80.5	360 ± 30	1456–1529 (45.5%) 1540–1635 (50.0%)	Charcoal
9	Poz-152863	90.5	325 ± 30	1483–1642 (95.4%)	Charcoal
10	Poz-152864	94.5	695 ± 30	1270–1316 (69.4%) 1360–1388 (26.1%)	Charcoal

### Bulk density and carbon accumulation

For each of the 91 samples, the water content in the wet sample (WC, %), organic matter content in the dry sample (ORG, %), ash content (ASH, g), ash-free bulk density (BD, g/cm^3^), peat accumulation rate (PAR, cm/yr), and peat carbon accumulation rate (PCAR, gC/m^2^/yr) were calculated. The volume of each sample was accurately measured using calipers. Each sample was then placed in separate crucibles, weighed, dried, and weighed again to determine the percent of WC. The dried samples were burned in a muffle furnace at 550 °C for 12 h and reweighed following the protocol by Heiri, et al.^112^ to determine ASH. BD was calculated by dividing the weight of the dry sample by the volume of the fresh sample and multiplied by ORG according to Chambers, et al.^113^. PAR was calculated based on the core chronology and then multiplied by the BD value obtained earlier and by 50% to obtain PCAR, following Loisel, et al.^114^.

### Plant macrofossils

The analysis of plant macrofossils was based on a modified protocol of Mauquoy, et al.^115^. Each sample of approximately 5 cm^3^ was wet sieved (mesh diameter: 200 μm). The content of monocot epidermis, mosses, leaf and needle remains, shrub branches, bark fragments, monocot and shrub roots was determined as percentages. Fruits, seeds, seed wing fragments, anthers, caryopses, spindles, scales, wholly preserved leaves and needles, sporangia, opercula, fungal sclerotia, and wood pieces were counted as total numbers in each sample. In addition, the percentage of non-identifiable organic matter (*Substantia humosa*) was also determined. Moss leaves (brown and *Sphagnum* mosses) were identified on slides using a magnification of × 200 and × 400. The classification of *Sphagnum* mosses was based on the publication of Laine, et al.^116^. All material was compared with several identification guides^116–120^. Insect remains (unidentifiable to the species level) were also found.

### Testate amoebae and quantitative reconstructions

Peat samples for testate amoeba analysis (size: 3 cm^3^) were washed under 0.3 mm sieves following a standard method^121^. Testate amoebae were analysed under a light microscope between × 200 and × 400 magnification until the sum of 100 tests per sample was reached^122^. Several keys and taxonomic monographs^123–125^ and online resources^126^ were used to achieve the highest taxonomic resolution. The results of testate amoeba analysis were used for the quantitative depth-to-water table (DWT) and trophy (pH) reconstructions. Both reconstructions were performed in C2 software^127^ using the European training set^128^.

### Pollen, spores, and non-pollen palynomorphs

Samples for palynological analysis (size: 1 cm^3^) were prepared using standard laboratory procedures^129^, followed by acetolysis. A single *Lycopodium* tablet (containing 18,407 spores per tablet; produced by Lund University) was added to each sample during the laboratory procedures for the calculation of microfossil concentration^130^. Pollen, spores, and selected non-pollen palynomorphs (NPPs) were counted under an upright microscope (Zeiss Axio SCOPE A1) until the number of total pollen sum (TPS) grains in each sample reached at least 500, apart from 13 samples in which pollen concentrations were very low. Sporomorphs were identified with the assistance of atlases, keys^131,132^, various publications^133,134^, and an image database of NPPs (https://non-pollen-palynomorphs.uni-goettingen.de). The results of the palynological analysis were expressed as percentages, calculations are based on the ratio of an individual taxon to the TPS, i.e., the sum of AP (arboreal pollen) and NAP (non-arboreal pollen), excluding aquatic and wetland plants (together with Cyperaceae and Ericaceae), cryptogams, and NPPs. The palynological diagrams were generated using Tilia software^135^.

### Charcoal

Microscopic charcoal particles (> 10 μm) were counted from depth-respective palynological slides until the combined total of charcoal particles and *Lycopodium* spores exceeded 200^136,137^. Sub-samples 14.5 and 38.5 cm were not analysed in this instance due to lack of appropriate material. Microscopic charcoal concentrations (particles/cm^3^) were calculated in relation to the number of *Lycopodium* spores counted in each sample. Microscopic charcoal influx or accumulation rates (MIC, particles/cm^2^/year) were calculated by multiplying microscopic charcoal concentrations by peat accumulation rates (PAR)^136^.

For macroscopic charcoal analysis, samples (size: 3 cm^3^) were sieved through a 0.5 mm mesh. To obtain a local fire signal, independent of the distal wind-blown fraction, only charcoal fragments exceeding 600 μm were analysed as part of this study^87^, using a binocular stereomicroscope with × 60 magnification. Whenever possible, macroscopic charcoal morphotypes (wood, grass, or leaf) were determined following a standard methodology^69,138,139^. Macroscopic charcoal concentrations (particles/cm^3^) were calculated by dividing the charcoal sum by sample volume. Macroscopic charcoal influx or accumulation rates (MAC, particles/cm^2^/year) were calculated using the macroscopic charcoal concentrations and PAR.

Scanning electron microscopy (SEM) and binocular stereomicroscopy was applied to the largest charred wood fragments (sampled at 86.5 and 85.5 cm) to assess taxonomy, micromorphology, and evidence of biological alteration. The morphology and chemical compositions were studied using an S-3700N Hitachi scanning electron microscope (SEM) coupled with a Thermo Scientific Energy Dispersive Spectrometer (EDS) detector and the Noran System 7 (NSS) analytical software. The observations were carried out at the Faculty of Geographical and Geological Sciences, Adam Mickiewicz University, Poznań, Poland, using BSE mode at an accelerating voltage of 20–30 kV, a working distance of 10 mm and a vacuum of 15–30 Pa.

#### Raman spectroscopy

The carbonaceous microstructures of macrocharcoal fragments, isolated from the peat monolith sub-samples (∑14) between 81.0 and 95.0 cm, were analysed for this study by applying µ-Raman spectroscopy (‘Raman’ *herein*). This was with the intention of assessing energy-dependent physiochemical changes that occurred during the formation of the charcoal in historic Stawek peatland wildfires, as a proxy for wildfire intensity established in Mauquoy, et al.^76^, Theurer, et al.^74^, Theurer, et al.^75^, and Theurer, et al.^77^.

To ensure all Raman spectra were robust, repeatable, and temporally comparable, charcoal sub-samples did not undergo any form of chemical preparation or treatment (e.g., bleaching), the influence over the carbonaceous microstructure, of which, remains uncharacterised. Instead, charcoals were isolated manually via randomised selection under a binocular stereomicroscope, ensuring a variety of fragment sizes and morphologies were analysed, and the inherent heterogeneity of natural fire activity was accounted for in selection.

During spectroscopic analysis, individual surface measurements (i.e., spectra) were spot-collected at random from up to 25 charcoal fragments per sample, where possible. Sites were selected for spectral acquisition according to their evident cellular structure and reflectivity, indicative of flat surfaces under incident light microscopy that are particularly conducive for a balanced signal–noise ratio.

Spectra were collected using a *Renishaw* ‘InVia Reflex’ Raman spectrometer at the University of Aberdeen. A green diode-pumped 514.5 nm laser (VIS, 24,000 l/mm) was applied through a *Leica* DM2700M incident light microscope, implementing × 50 objective magnification. Applied power did not exceed 0.3 mW (1% total output). This remains in accordance with protocols established to preserve sample integrity^140^. Calibration to a silicon reference peak (~ 520.5 cm^−1^) was conducted prior to spectral acquisition, and reference offsetting utilised as and when appropriate. A laser spot size of 1–2 µm was applied during acquisition. Spectra were generated between ~ 1100–1700 cm^−1^, centred at ~ 1400 cm^−1^, comprising three consecutive accumulations over 15 s total exposure (5 s/accumulation) at a scan resolution of ~ 3 cm^−1^. A cosmic ray removal function was also applied during each acquisition.

In order to generate spectral band separation values (‘RBS’, cm^−1^) for charcoal materials, from which the measure of wildfire intensity is derived^77^, a standardised method of automated deconvolution was applied in this study. This coded method, incorporates a ‘peak fit’ base code^141,142^ into a (modified) secondary deconvolution code, as developed in Schito and Corrado^143^ and utilised in^144^. This was conducted within MATLAB (v. R2021a) and the code applies a smoothing function and linear baseline to input spectra, anchored between 1050 and 1150 cm^−1^ and 1650–1750 cm^−1^. Following this, two pseudo-Voigt (50% Gaussian–Lorentzian) bands shapes are fit automatically to Raman bands ‘D’ (~ 1350 cm^−1^) and ‘G’ (~ 1585 cm^−1^). From this, a series of parametric outputs are generated based on the band application, including Raman band separation (‘RBS’ *herein*) values. RBS, generated by the progressive separation of bands ‘D’ and ‘G’ with increasing thermal maturation of the carbon microstructure, has shown a consistent and robust direct proportionality with pyrolysis-intensity (Schito, et al.^145^ *and references therein*), as an (experimental) proxy for wildfire intensity^75^. Raman Band Separtation values, as derived here, have been applied to Eqs. (1) and (2) after Theurer, et al.^77^, enabling the reconstruction of equivalent values for ‘energy released’ (kJ) and ‘total incident energy’ (MJ) for fire activity in which this charcoal was generated. These values represent measures of true wildfire intensity^97^, corresponding to energy release and radiative absorption as experienced by vegetation under fire front progression^96,146^, respectively. The reader is directed to Theurer, et al.^77^ for further details as to the development of this reconstruction method.

1 $$Energy\, Released \left( {kJ} \right) = 3.448 \times \left[ {RBS} \right] - 621.762$$

2 $$Total\, Incident\, Energy \left( {MJ} \right) = 0.514 \times \left[ {RBS} \right] - 104.04$$

Acquisition and deconvolution protocols applied here remain consistent with established practices in the Raman spectroscopic study of charcoal (e.g^74–77,147–149^.,).

## Data Availability

The datasets generated and/or analysed during the current study are available in the Mendeley Data repository, https://data.mendeley.com/datasets/c43j5cm2c4/2.
